# Inflammasomes in *Mycobacterium tuberculosis*-Driven Immunity

**DOI:** 10.1155/2017/2309478

**Published:** 2017-12-04

**Authors:** Sebastian Wawrocki, Magdalena Druszczynska

**Affiliations:** Division of Cell Immunology, Department of Immunology and Infectious Biology, Institute of Microbiology, Biotechnology and Immunology, Faculty of Biology and Environmental Protection, University of Lodz, Banacha 12/16, 90-237 Lodz, Poland

## Abstract

The development of effective innate and subsequent adaptive host immune responses is highly dependent on the production of proinflammatory cytokines that increase the activity of immune cells. The key role in this process is played by inflammasomes, multimeric protein complexes serving as a platform for caspase-1, an enzyme responsible for proteolytic cleavage of IL-1*β* and IL-18 precursors. Inflammasome activation, which triggers the multifaceted activity of these two proinflammatory cytokines, is a prerequisite for developing an efficient inflammatory response against pathogenic *Mycobacterium tuberculosis* (*M.tb*). This review focuses on the role of NLRP3 and AIM2 inflammasomes in *M.tb*-driven immunity.

## 1. Introduction

*Mycobacterium tuberculosis* (*M.tb*), the causative agent of tuberculosis (TB), is a facultative intracellular bacterium that can survive and replicate within host macrophages [1, 2]. By avoiding critical components of macrophage-killing repertoire such as phagosome-lysosome fusion, phagosome acidification, activity of lysosomal enzymes or reactive oxygen, and nitrogen intermediates, *M.tb* evades killing and eradication [3]. In addition to phagocytic activity and ability to present antigens to T-cells, macrophages are key cells that regulate the antimycobacterial immune response via secreted cytokines. The functional capacity of macrophages in fighting infection depends on the degree of their activation. Inactive macrophages have limited ability to inhibit the growth of ingested mycobacteria, thereby serving as a safe life niche. After activation by interferon-gamma (IFN-γ) that is secreted by T-cells, macrophages acquire enhanced bactericidal strength enabling them to kill mycobacteria growing intracellularly [4]. The IFN-γ-driven antimicrobial properties of phagocytes are augmented by IL-18 and IL-1*β*, two proinflammatory cytokines processed by caspase-1 that are recruited to the inflammasomes, multiprotein platforms composed inter alia of intracellular sensors for pathogen- or host-derived molecules. IL-18, belonging to the IL-1 family, is produced by a wide range of immune and nonimmune cells [5–7]. The IL-18 precursor (pro-IL-18) is converted by caspase-1 into an active molecule, which forms a signaling complex with IL-18R [8, 9]. The receptor is composed of two chains: alpha (IL-18Ra) and beta (IL-18Rb). IL-18Rb is a signal transduction chain, essential for the formation of a high affinity complex and cell activation. The primary role of IL-18 is to induce IFN-γ production in cooperation with IL-12 or IL-15, although immunological effects exerted by IL-18 are dependent on the cytokine microenvironment. IL-18 is able to polarize T lymphocyte response towards Th1, induce T-cell proliferation, activate NK cells, enhance CD8(+) T cytolytic activity, and augment, apart from IFN-γ, the production of varied cytokines including tumor necrosis factor-α (TNF-α), interleukin- (IL-) 4, IL-5, IL-13, IL-17, and granulocyte-macrophage colony stimulating factor (GM-CSF) [8, 10, 11]. Thus, the multifaceted activity of IL-18 seems to play a prominent role in host defense against both extracellular and intracellular pathogens, including *M.tb*. However, an excessive IL-18 response might contribute to the induction of pathomechanisms leading to the damage of cells and tissues [12, 13]. Therefore, the proinflammatory activity of IL-18 is balanced by a constitutively secreted IL-18 binding protein (IL-18BP), whose binding to IL-18 decreases the production of IFN-γ and other cytokines, thereby reducing the risk of immunopathology [14]. The other inflammasome-dependent cytokine, IL-1*β*, which is mainly produced by monocytes and macrophages, plays an important role in inflammation and host immune response by affecting the function of various cells, either alone or in combination with other cytokines [15–17]. The activity of IL-1*β* is tightly regulated at the levels of its transcription and release. The production of IL-1*β* is regulated by several proteins including pyrin, PI-9 (the caspase-1 inhibitor proteinase inhibitor 9), and some CARD-containing proteins, which interfere with the recruitment of caspase-1 or directly neutralize its activity [18]. The effects of IL-1*β* are exerted via binding specific cell surface receptors—IL-1RI and IL-1RII [19]. As in the mature IL-18 form, active IL-1*β* is created after the proteolytic cleavage of its precursor by inflammasome-dependent caspase-1. Mature IL-1*β* plays important homeostatic functions in organisms and is implicated in the initiation of antimicrobial immunity via the induction of TNF-α and IL-6 release and polarization of Th17 response, which improve protective mucosal host defense by the secretion of IL-17 and IL-22 [20, 21]. The proinflammatory role of IL-1*β* in the resistance against *M.tb* has been confirmed by the observation that IL-1*β* or IL-1R knockout mice were found to be more susceptible to TB showing high mortality and increased bacterial burden in the lungs [22]. Additionally, double-deficient IL-1α/*β* mice had significantly larger granulomas, and their alveolar macrophages produced less nitric oxide than the cells from wild-type animals [23].

## 2. Inflammasomes—Mediators of Inflammation

Inflammation is an evolutionarily conserved protective response to noxious stimuli mounted by the innate immune system of the host. Immune deficiencies leading to insufficient development of inflammation processes may result in severe and recurrent infections, although overly intense activation of the inflammation cascade may be a cause of chronic systemic inflammatory disorders [24, 25]. The development of innate immunity starts from the recognition of conservative antigenic structures called DAMPs (danger-associated molecular patterns) and PAMPs (pathogen-associated molecular patterns) by pattern recognition receptors (PRRs) presented on the surface of first-line defense immune cells—macrophages and neutrophils. Activation of these receptors triggers a cascade of signals that results in the induction of multiple proinflammatory cytokines. The final step of the activation is the production of oxygen and nitrogen radicals, essential elements of the intracellular killing system. The secretion of these radicals is under strict control of a variety of monocyte/macrophage-derived cytokines such as IL-1*β* and IL-18. The key role in this process is played by structures called inflammasomes, multimeric protein complexes that control many aspects of innate and adaptive immunity. Through their cooperation with PRRs, inflammasomes activate host defense pathways resulting in clearance of various viral and bacterial infections, including those caused by mycobacteria. They function as an activating scaffold for inflammatory caspases that play an essential role in the maturation and secretion of proinflammatory cytokines as well as in pyroptosis, an inflammatory death of infected cells [26, 27]. Caspases are produced as inactive proenzymes that dimerize and undergo cleavage to form active molecules. Assembly into dimers, facilitated by various adaptor proteins binding to specific regions of their precursor forms—procaspases, is achieved through inflammasome formation [28]. Activated inflammatory caspases, typically caspase-1, lead to the generation of active IL-1*β*, IL-18, and IL-33 from their proprotein precursors. The mature cytokines are engaged in the recruitment of immune cells to the sites of infection and enhancement of the host's defensive responses against invading pathogens [26].

The inflammasomes are activated by multiple recognition receptors, which determine their structure and function. The canonical inflammasome sensors are nucleotide-binding domain–like (NLR) proteins and absent in melanoma 2–like (ALR) proteins and PYRIN. All of them have the ability to assemble inflammasomes and activate the inflammatory caspase-1.

The NLR family contains the NLRPs (or NALPs) and the IPAF (ICE-protease-activating factor) subfamilies [29, 30]. Each NLR molecule (NLRP1, NLRP3, NLRP6, NLRP7, NLRP12, or NAIP/NLRC4) recognizes specific ligands that activate the assembly of the inflammasome. NLR proteins consist of the conserved nucleotide-binding and oligomerization domain (NACHT or NOD), an N-terminal caspase recruitment domain (CARD) or pyrin domain (PYD) or baculovirus inhibitor repeat- (BIR-) like domain, and C-terminal leucine rich repeats (LRRs) [26, 31–35]. LRRs are responsible for the recognition of PAMPs, while the NACHT domain activates proinflammatory cytokine pathways via ATP-dependent oligomerization [26, 29]. The NLRP1 inflammasome has a CARD that activates caspase-1 [36, 37], and therefore the recruitment of ASC is not required to interact directly with procaspase-1. However, it has been shown that the participation of ASC in the process enhanced the activation of the enzyme. In contrast, NLRP3 contains no typical CARD domain that contributes to the activation of caspase-1 through the interaction of the PYD domain of NLRP3 with ASC [25]. Compared with NLRP1 and NLRP3, the IPAF protein does not contain a PYD but instead has a CARD that interacts directly with procaspase-1 without the need for ASC [38].

The members of the ALR group (known as the PYHIN family) are characterized by the presence of the pyrin domain (PYD) and one or two hematopoietic IFN-inducible nuclear antigens with 200 amino acid repeat (HIN-200) domains [26]. The PYD recruits proteins for the formation of inflammasomes, while the HIN domain recognizes and binds to DNA that can be found in the cytosol [26]. The best-known ALRs, absent in melanoma 2 (AIM2) and IFN-γ inducible protein 16 (IFI16), function as intracellular immune sensors that detect microbial DNA. The PYHIN proteins differ in their localization in the cell compartments; AIM2 can be found in the cytosol, whereas IFI16 is usually localized in the nucleus [39].

PYRIN, another canonical inflammasome-activating protein, is composed of an N-terminal PYD followed by two central B-box zinc finger and coiled-coil domains and in humans, a C-terminal B30.2/rfp/SPRY domain [40]. PYRIN associates through a PYD-PYD interaction with ASC protein, leading to its oligomerization that results in caspase-1 activation and interleukin-1*β* processing [40]. The activation of the PYRIN inflammasome is induced by the inactivation of RhoA GTPase by bacterial toxins [26, 41]. The process of activation has been detected in both mice and humans, suggesting that the B30.2/rfp/SPRY domain is not necessary for its initiation.

## 3. Inflammasomes in *Mycobacterium tuberculosis* Infection

**Figure 1:**
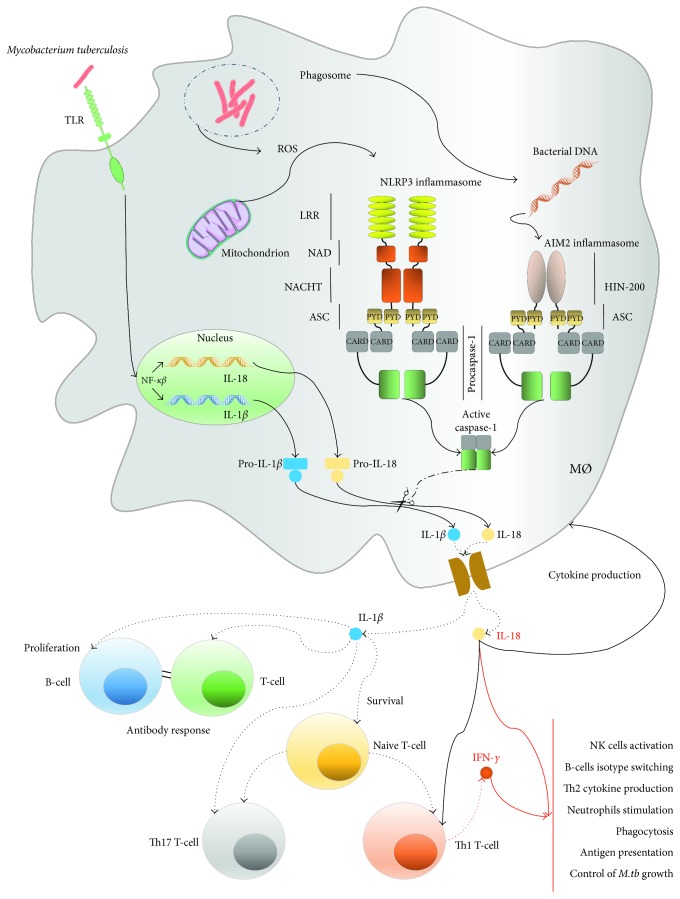
AIM2 and NLRP3 inflammasome activation pathways induced by *Mycobacterium tuberculosis*.

The inflammasomes have been found to play important roles in host immunity against mycobacteria since it has been found that mice deficient in IL-18, IL-1*β*, or IL-1 receptor type I (IL-1R1) are more susceptible to *M.tb* infection [42–46]. Two inflammasomes, containing NLRP3 and AIM2 molecules as sensor proteins, were found to play a crucial role in *M.tb*-induced immunity (Figure 1) [20, 47, 48].

The NLRP3-containing inflammasome can be activated by a wide group of stimuli including whole mycobacterial cells, as well as viruses, fungi, environmental chemical irritants, and host-derived molecules such as extracellular ATP, fibrillar amyloid-*β* peptide, and hyaluronan [22, 49–53]. The NLRP3 inflammasome-activated responses result in the release of significant amounts of caspase-1, which leads to maturation and secretion of IL-1*β* and IL-18 and activation of pyroptosis [26]. The process of NLRP3 activation is triggered by at least two signals: (1) a priming signal eliciting the expression of NLRP3, pro-IL-1*β*, and pro-IL-18 genes after TLR stimulation and (2) an activation signal leading to the autocatalytic activation of procaspase-1 and proteolytic cleavage of pro-IL-1*β* and pro-IL-18. In most cell types, NLRP3 priming is a prerequisite for deubiquitination and assembly of the NLRP3 inflammasome. Relocalization of NLRP3 to the mitochondria is followed by the secretion of mitochondrial factors into the cytosol, potassium efflux through membrane ion channels, and release of cathepsin resulting in destabilization of lysosomal membranes. Apoptosis-associated speck-like protein (ASC) plays an important role in the formation of an effective inflammasome. ASC recruits procaspase-1 through its C-terminal caspase recruitment domain (CARD) and interacts with NLRP3 via its pyrin domain (PYD), serving as a bridge between these two molecules. The autocatalysis of procaspase-1 results in its cleavage and transformation into active caspase-1, which in turn cleaves the precursors of two proinflammatory cytokines, IL-1*β* and IL-18, leading to their secretion into the cytoplasm or induction [24, 25, 48, 54, 55]. However, the mechanism of triggering the NLRP3 inflammasome complex activation cascade is still a subject of debate, and at least three models for the process have been proposed. The first suggestion is that the activation mechanism is associated with an efflux of potassium ions out of the cell and a reduction in their intracellular concentration. Such a model of activation occurs in monocytes/macrophages after stimulation with numerous stimuli including ATP, nigericin, bacterial cells, or their components [56, 57]. Recently, NEK7 protein, a member of the family of NIMA-related kinases (NEK proteins), has been identified as an NLRP3-binding protein that acts downstream of potassium efflux to regulate NLRP3 assembly and activation [58]. He et al. demonstrated that in the absence of NEK7, caspase-1 activation and IL-1*β* release were abrogated in response to signals that activate NLRP3 [58]. According to the second suggested mechanism, inflammasome activation is a result of lysosomal membrane damage and release of the phagosome content into cytosol [22, 59]. The third and most accepted model assumes that the induction of the NLRP3 inflammasome complex is caused by mitochondrial reactive oxygen species (ROS) [60–63]. The common final step in all of these models is the release of cathepsins into the cytosol leading to the lysosomal destabilization and conversion of procaspase-1 into a biologically active caspase-1 form. It should also be mentioned that formation of the NLRP3 inflammasome and cytokine release occur independently of transcriptional upregulation [64]. Juliana et al. showed that TLR4 signaling through MyD88 nontranscriptionally primed the NLRP3 inflammasome by its deubiquitination. The mechanism was dependent on mitochondria-derived reactive oxygen species and was involved in the secretion of cytokines, such as IL-18, and other inflammatory mediators such as high-mobility group protein 1 (HMGB1) [64, 65].

The AIM2 (absent in melanoma 2) receptor, possessing a C-terminal HIN-200 domain and an N-terminal pyrin domain (PYD), triggers AIM2 inflammasome activation, inflammatory cell death (pyroptosis), and release of IL-1*β* and IL-18 in response to cytosolic double-stranded (ds) DNA [66, 67]. Studies of gene-targeted AIM2-deficient mice have shown that AIM2 inflammasomes play a role in host defense against viruses and intracellular bacterial pathogens such as listeriae and mycobacteria [68–70]. AIM2 inflammasomes can be activated by DNA sequences having at least 80 base pairs in length in a sequence-independent manner [71, 72]. The HIN-200 and PYD domains take part in forming a complex, which is maintained in an inactive state during homeostasis [71, 73]. Binding of dsDNA to HIN-200 facilitates oligomerization of AIM2, and the resulting conformational change exposes the N-terminal PYD to allow the recruitment of the adaptor protein ASC. The CARD of ASC binds the CARD of procaspase-1, that forms an active AIM2 platform. Upon autoactivation, caspase-1 directs maturation and secretion of proinflammatory cytokines [48, 55, 66, 68, 74].

The latest data suggest that NLRP3- or ASC-deficient animals are characterized by impaired inflammasome formation and increased susceptibility to TB [20, 54, 68, 75, 76]. However, NLRP3^−/−^ and ASC^−/−^ mice produced IL-18 and IL-1*β* levels comparable to those of wild-type mice, which suggests the involvement of inflammasome-independent pathways in the secretion of these cytokines [21, 42, 47]. Many reports have demonstrated that a wide range of microorganisms are able to inhibit inflammasome activation and function. Viruses and many bacterial pathogens develop several mechanisms of repression of inflammasome folding; however, not all mechanisms are clearly understood. *Yersinia enterocolitica* produce YopE and YopT proteins that supress caspase-1 maturation, whereas YopK protein of *Y. pseudotuberculosis* binds to the type III secretion system, thereby preventing the recognition of the pathogen by host cell inflammasome. *Pseudomonas aeruginosa* mediates suppression of NLRC4-inflammasome by secreting ExoU and ExoS effectors, whose mechanism of action still needs elucidation. Virulent *M.tb* can inhibit the formation of AIM2 and NLRP3 inflammasomes both directly and indirectly, but the factors responsible for the inhibition have not been recognized thus far. One of the likely mechanisms is the activity of Zn-metalloprotease called ZMP1, which inhibits the activation of NLRP3 inflammasome and, as a consequence, leads to the reduction of caspase-1 activity [77–79]. Master et al. showed that infection of mice macrophages with zmp1-deleted *M.tb* induces activation of the inflammasome, resulting in enhanced maturation of phagosomes, increased IL-1*β* secretion, and better *M.tb* clearance in lungs [79]. It is probable that *M.tb* is able to restrain the activation of other inflammasome types, but evidence is needed to confirm this hypothesis. In addition to the induction of inflammasome activation via PRRs, *M.tb* antigens can modulate other innate immunity-associated functions. One recently identified protein, tyrosine phosphatase (Ptp) A, enters the nucleus of the host cells and regulates the transcription of many host genes involved in the mechanisms of innate immunity, cell proliferation, and migration [80]. The enzyme is also able to dephosphorylate certain host proteins (p-JNK, p-p38, and p-VPS33B), leading to inhibition of phagosome-lysosome fusion and blocking the acidification of phagosomes. Both activities are crucial for *M.tb* virulence in vivo through the promotion of *M.tb*'s intracellular survival in macrophages [80]. *M.tb* often escapes from the phagosome within a few days of the invasion of the host organism and creates difficulties in assessing the potential role of inflammasomes during the initial stages of mycobacterial infection. Moreover, the evaluation of IL-1*β* and IL-18 produced as a result of inflammasome activation is inadequate in revealing the significance of formed multiprotein platforms in the course of developing infection. The initiation of phagocytosis causes a decrease in the levels of potassium ions in macrophages, which have been found to be one of the crucial inflammasome activators during infections with *M.tb* and nontuberculous mycobacteria [81]. Other regulators such as thioredoxin-interacting proteins, activated by the increase in reactive oxygen species in cytosol, are thought to have minor effect on the formation of inflammasomes in *M.tb* infection [47]. The signaling cascade can also be activated by the mycobacterial type VII secretion system (ESX-1), which is responsible for translocation of extracellular DNA (eDNA) in cytosol and the production of IFN-*β*. Many studies have demonstrated that, at the molecular level, IFN-*β* regulates the AIM2 inflammasome activity [82, 83]. Some ESX-1-deficient *M. smegmatis* mutants have been shown to possess limited capacity for AIM2 inflammasome activation. However, in contrast to nontuberculous mycobacteria (NTM), *M.tb* mutants lacking ESX-1 system failed to inhibit AIM2 formation, while the wild-type strain inhibited the inflammasome activation [47, 84]. The suggested mechanism of inhibition involves the IFN-*β*-mediated induction of IL-10, which in turn suppresses IL-1*β* production [85, 86]. However, further investigation is needed to elucidate the molecular mechanism of *M.tb*-driven AIM2 inhibition and its consequences for bacterial virulence. *M. bovis* BCG vaccine strain, which does not possess the ESX-1 system, poorly activates multiple NLR and inflammasome complex components including caspase-1 [87]. The bacilli repress the expression of thioredoxin-interacting protein (TXNIP), an antioxidant inhibitor recruiting caspase-1 to the NLRP3 inflammasome. The inhibition of TXNIP by BCG limits NLRP3 activation and restrains pyroptosis following mycobacterial infection. Proinflammatory responses to BCG bacilli was found to be driven primarily through Toll-like receptors (TLRs), since BCG does not activate expression of genes downstream of TLR/MyD88- and NOD-2-driven NF-*κβ* and AP-1 pathways. However, BCG is still able to induce moderate IL-1*β* secretion as measured by transcription of inflammasome network genes [87, 88]. Understanding BCG-induced pathways of inflammasome activation can be helpful in improving the existing vaccine or developing new antituberculous vaccines. The recombinant BCG ΔureC::hly vaccine candidate (VPM1002) has been shown to induce improved protection against TB over the parental BCG strain [4]. Saiga et al. demonstrated that VPM1002 activated the AIM2 inflammasome and caspase-1 through the ability of listeriolysin to perforate phagosome membranes, which is encoded by the *hly* gene integrated into BCG genome [4]. The perforation facilitates the release of mycobacterial DNA into the cytosol, in a way that is similar to the ESX-1 system of *M.tb*. Mice vaccinated with VPM1002 showed increased production of IL-1*β* and IL-18 as well as induction of the stimulator of IFN genes (STING)-dependent autophagy, which promotes delivery of BCG antigens to MHC molecules and improves their presentation to T-cells [4].

Apart from direct induction of proinflammatory cytokine secretion, the activated caspase-1 triggers the pyroptotic death of infected cells. The cytosolic protein Gasdermin D (GSDMS) is a key mediator of this process. The cleavage of GSDMD by activated caspase-1 results in the release of its N-terminal fragment (GSDMD-NT), which forms pores in the plasma membrane of the infected cell leading to the elimination of the pathogen [26, 89–91]. The pores disrupt cell membrane integrity allowing water influx, cell swelling, and osmotic lysis together with an efflux of small molecules, including proinflammatory cytokines. GSDMD-NT is able to kill both cell-free and intracellular microorganisms and can be thought as a new antibacterial agent. However, it is still not known whether GSDMD-NT is able to permeabilize the membrane of the phagosomes and kill the bacteria hidden within these organelles. So far, there is no evidence of such a function. It is probable that the inhibition of bacterial growth is mediated by other caspase components. Using single-cell analysis, Thurston et al. demonstrated that the replication of cytosolic *Salmonella typhimurium* was inhibited independently or prior to the onset of cell death, suggesting that caspase-1 and caspase-11 might have additional functions in the elimination of cytosolic bacteria [92].

## 4. Therapeutics Targeting Inflammasome Pathways

Biologic agents interfering with inflammasome activation may provide new means of therapeutical interventions for many diseases. These agents may target either upstream processes of inflammasome regulation or downstream IL-1 signaling [41]. Inappropriate activity of inflammasomes has been found to be involved in the pathogenesis of certain autoinflammatory skin disorders such as cryopyrin-associated periodic syndrome (CAPS) or familial Mediterranean fever (FMF) as well as a number of chronic inflammatory diseases such as multiple sclerosis, gouty arthritis (gout), atherosclerosis, type 2 diabetes, and obesity [29, 93, 94]. Moreover, mechanisms controlling the NLRP3 inflammasome arrangement have also been implicated in the development of lung, kidney, and liver diseases [95–97]. Colchicine, a drug used for treatment of gout, has been shown to inhibit macrophage NLRP3 inflammasome assembly and activation in vitro and in vivo [98]. Colchicine blocks monosodium urate crystal-induced NLRP3 inflammasome-driven caspase-1 activation and IL-1*β* processing and release, suppresses the expression of genes involved in cell regulation, and inhibits IL-1-induced L-selectin expression on neutrophils [99]. Other therapeutics that target inflammasome-driven end products include VX-765 (inhibitor of caspase-1 activation), Anakinra (recombinant form of IL-1 receptor antagonist), Canakinumab (monoclonal antibody against IL-1*β*), Rilonacept (IL-1 inhibitor), IL-18 binding protein, and anti-IL-18 receptors antibodies [8, 41, 100]. A number of new molecules have been identified as inhibitors of IL-1*β* processing (glyburide, parthenolide, CRID3, auranofin, isoliquiritigenin, *β*-hydroxybutyrate, and MCC950); however, confirming their clinical utility will require additional time and research [24].

## 5. Conclusion

Inflammasomes have been implicated as specialized signaling platforms critical for the regulation of both innate immunity and inflammation. *M.tb* has been shown to modulate the host innate immune response by delaying cell death systems of the host, thereby facilitating its own proliferation. Understanding the molecular mechanisms of inflammasome activation during intracellular pathogen infections such as with *M.tb*, and the evasive mechanisms employed by this evading pathogen, may lead to development of more potent therapies to combat the proliferation of *M.tb*.
